# Effect of Mo Content on the Structural, Mechanical, and Tribological Properties of New Zr-Nb-Mo Alloys Obtained by Combining Powder Metallurgy and Vacuum Arc Melting Methods

**DOI:** 10.3390/ma17143483

**Published:** 2024-07-14

**Authors:** Julia Zając, Izabela Matuła, Adrian Barylski, Krzysztof Aniołek, Marcin Nabiałek, Julia Flesińska, Grzegorz Dercz

**Affiliations:** 1 Institute of Materials Engineering, University of Silesia in Katowice, 75 Pułku Piechoty Street 1 A, 41-500 Chorzów, Poland; izabela.matula@us.edu.pl (I.M.); adrian.barylski@us.edu.pl (A.B.); krzysztof.aniolek@us.edu.pl (K.A.); julia.flesinska@op.pl (J.F.); 2 Department of Physics, Częstochowa University of Technology, 19 Armii Krajowej Av., 42-200 Częstochowa, Poland; marcin.nabialek@pcz.pl

**Keywords:** zirconium alloys, biomaterials, arc melting, powder metallurgy

## Abstract

Considering the high demand for innovative solutions in medicine, a major increase in interest in biomaterials research has been noticed, with the most significant advancements in metals and their alloys. Titanium-based alloys are one of the most recognised in the scientific community but do not represent the only way to achieve optimal results. Zirconium alloys for medical applications are a novelty with significant research potential based on their outstanding properties, which may be of value for medicine. The aim of the present study was to obtain new biomedical Zr-Nb-Mo alloys with varying ratios of their respective elements—Zr and Mo—using combined powder metallurgy (PM) and arc melting (VAM) methods. The obtained samples underwent microstructure analysis using an optical microscope (OM) and a scanning electron microscope (SEM). The study of element distribution was conducted with energy dispersive spectroscopy (EDS), whereas the phase composition was determined using X-ray diffraction (XRD). Mechanical properties were examined with a Micro Combi Tester MCT^3^, whereas tribological properties were assessed with a TRN Tribometer, and Ringer’s solution was used as a lubricant. Additionally, the wear tracks of the studied samples were observed using the SEM. The research results indicated that increased Mo content conduced to microstructure refinement and homogeneity. Furthermore, the higher content of this element contributed to the growth of the HV_IT_, H_IT,_ and E_IT_ parameters, together with the improvement in the tribological performance of the alloys. XRD analysis revealed that the obtained samples were multiphase, and raising the Mo addition promoted the formation of new phases, including a ternary phase—Zr_0.9_Nb_0.66_Mo_1.44_ (Fd$\bar{3}$m). The chemical composition study showed uneven distribution of niobium and areas of uneven mutual distribution of zirconium and molybdenum.

## 1. Introduction

According to global trends, medicine is one of the world’s fastest-growing economic sectors. The constantly increasing life expectancy of the population is a significant driver of market growth. The international implants market was valued at USD 10.4 trillion in 2023. It is estimated to reach USD 16 trillion by 2029—an increase of 7.5% [1]. Significant advances in biomaterials research serve not only to improve patients’ quality of life but also to facilitate the work of clinicians and develop treatments based on individual variability, so-called personalised therapy. In this respect, metals and their alloys—currently the most common materials used in medicine—have the greatest research potential. According to economic forecasts, medical metallic alloys used in orthopaedics and cardiac surgery will reach a global value of USD 17.6 trillion in 2024 [2]. Metals are used as structural materials for instrumentation and implants in hard tissue and cardiac surgery. Significant advances in research into metallic biomaterials are due to the broad possibilities of modifying their properties and their wide availability. Despite their excellent characteristics, medical alloys still carry the risk of triggering adverse effects associated with, among other things, abnormal bone biomechanics, corrosion processes, and harmful alloying additives. Materials based on titanium, characterised by high biocompatibility, are of significant interest [3,4]. Zirconium-based alloys with niobium and molybdenum additives offer interesting physicochemical properties, including biofunctional properties. Zirconium, niobium, and molybdenum are mainly known as alloying additives to commonly used titanium alloys in the metallic biomaterials industry. Still, in the form of a stand-alone alloy, they are a novelty with a decidedly high research potential [4,5,6] because the unique properties of Zr-based alloys, after possible surface modification, offer enormous and beneficial potential for the corrosion resistance of an implant. Thus, with the studies in this article, the authors contribute to future expectations for the design and manufacturing of new materials for medical applications.

The proposed production methods—powder metallurgy and vacuum arc melting—are known in both the biomedical and zirconium industries [3,6,7]. The application of each method leads to different properties of a material; for example, powder metallurgy leads to the development of a porous structure in the alloy, while VAM mostly favours the bulk material. Both may be beneficial, but for some metals, a satisfactory result requires more. Combining them together might be technologically challenging. However, it eliminates their individual disadvantages and results in a high-quality alloy meant for demanding applications. Powder metallurgy allows for both a thorough homogenisation of the initial powders and initial mechanical synthesis, leading to a micro “master alloy”. Consequently, this preparation of green compacts, together with subsequent arc melting, leads to an alloy with high purity and exquisite mechanical properties.

### 1.1. Zirconium Properties and Potential

Zirconium is a transition metal with a typically metallic appearance, sheen, and ductility. Zr is characterised by high strength at a relatively low density (6.51 g/cm^3^); its elastic modulus reaches approximately 100 GPa. It is chemically stable and has a melting point of 1852 °C. Zirconium exhibits high corrosion resistance in aggressive environmental conditions, i.e., salty sea water, strong alkalis and salts, and dry chlorine [7,8]. It is widely used in metallurgy as an alloying additive meant to increase toughness and impact strength or improve a material’s heat and corrosion resistance, i.e., steel or aluminium alloys. It also has excellent deoxidising properties and positively affects the durability of coatings, increasing their hardness and resistance to abrasive wear [7,9,10]. Most importantly, zirconium particles are environmentally harmless, and their materials can be recycled [11,12].

Zirconium-based alloys are the most prevalent alloys in the nuclear industry, and their unique properties described earlier are the primary reason for their use in this demanding sector. The common features of these materials are their anisotropy, lack of response to irradiation, low active cross-section, and very high corrosion and erosion resistance, achieved, among other things, through zirconium’s high affinity to oxygen. Alloys like Zircaloy-2 and Zircaloy-4 are used as fuel rod cladding and structural materials for high-pressure nuclear reactors (i.e., BWR, PWR) [7].

In comparison with titanium, metallic zirconium is an even weaker electrical conductor, showing minimal susceptibility to a magnetic field. Therefore, it is not a thrombotic agent and does not pose a risk of stopping blood circulation. In addition, the paramagnetism of zirconium alloys reduces the risk of image artefacts during examinations with medical devices, i.e., magnetic resonance [13,14].

Zirconium exhibits a low level of cytotoxicity and has no mutagenic or carcinogenic effects. Zr is remarkable for its bioactivity, promoting bone regeneration and growth processes. Studies have shown that, for both cytotoxicity and osteointegration, the properties of zirconium are superior to those of commonly used titanium [14,15].

A considerable proportion of the inflammatory reactions occurring in the human body after implantation are caused by wear and corrosion, which result in metal ions penetrating tissues and body fluids. Minimising the migration of particles from the implant into the body is of significant importance, keeping in mind that their type is also relevant. Metal ions, e.g., aluminium, vanadium, chromium, or nickel, cause more toxic reactions than niobium, zirconium, or molybdenum ions [16,17]. Zirconium is a metal with excellent corrosion resistance, and, because of its ability to self-heal, its surface is covered by a layer of inert zirconium oxide, providing additional protection against wear [8].

In light of the high demand for a new type of material for potential medical applications, highly biocompatible zirconium is proposed. The development of process parameters for the arc melting of zirconium-based alloys is challenging because of the high melting point and susceptibility to oxidation. The main aim of this work is to produce and test the effect of varying molybdenum content, with constant niobium content, on the structure, microstructure, and mechanical properties of the new Zr-Nb-Mo alloy.

### 1.2. The Role of Alloying Elements—Niobium and Molybdenum

Introducing alloying additives aims to obtain new or improved properties of a pure metal. For biomaterials, the beneficial effects on biocompatibility and bioactivity are also of particular importance. It is essential that none of the alloying elements, nor the compounds that result from their correlation, induce adverse reactions in the organism. At the same time, they should contribute to obtaining a homogeneous material with a stable phase structure, high corrosion resistance, and adequate mechanical performance.

Niobium is a non-mutagenic, non-carcinogenic element with an exceptionally low cytotoxicity index. It exhibits an impressive bioactivity, similar to zirconium, which promotes the osteointegration process of the implant [15,17]. When paired with zirconium, niobium enhances the strength and corrosion resistance of an alloy, mainly caused by hydrogen intake. Doping Zr with niobium allows alloys to maintain ductility while improving the tensile strength and yield strength of the material. Nb also serves as a β-Zr stabilizer, lowering the temperature of the α→β phase transition [7].

According to the latest studies, molybdenum is a biocompatible element [17,18]. Its presence in zirconium alloys significantly influences their mechanical properties, increasing tensile strength and yield strength because of solid solution reinforcement and inhibition of the Zr grain growth, resulting in microstructure refinement [13,19]. In addition, a properly heat-treated Mo-doped zirconium alloy exhibits an increase in hydrogenation and corrosion resistance, including nodular corrosion [7].

## 2. Materials and Methods

The studied material consists of commercially available Atlantic Equipment Engineers’ (AEE, New York, USA) powders of Zr (ZR-105, purity > 99%, particle size of 20 ± 60 mesh), Nb (CB-101, purity of 99.8%, particle size of 1–20 µm) and Mo (MO-101, purity of 99.98%; particle size of 1–2 µm). Eight chemical compositions with varying Zr and Mo and constant Nb contents were prepared (Table 1).

The first material preparation step was pre-synthesis and homogenization in a planetary ball-mill Fritsch Pulverisette 7 premium line (Weimar, Germany) under a protective gas atmosphere (Argon 5.0). The milling media and bowls were made of hardened stainless steel. The following parameters were used: rotation speed of the bowl of 100 rpm, milling time of 72 h, and the weight ratio of the powder to the balls of 1:5. After homogenisation, the material was subjected to cold isostatic pressing under 450 MPa. The next stage was the melting of green compacts in a vacuum arc furnace (VAM) in an Ar-protective atmosphere at a pressure of 1.2 bar. Prior to the melting process, a high-purity Ti-getter was used to remove residual gases from the chamber. The obtained buttons were flipped over and remelted five times for 60 s to homogenise the alloy. Lastly, the materials were kept in the induction furnace at 1000 °C for 24 h.

For further analysis, the specimens were prepared using a standard metallographic procedure as follows: grinding using abrasive papers and polishing by rotary discs covered with felt moistened with Struer’s colloidal OP-S silica suspension (Copenhagen, Denmark) with a grain size of 0.04 µm. Etching was performed in a reagent consisting of 45 mL 90% lactic acid, 45 mL 65% nitric acid (V), and 8 mL 40% hydrofluoric acid.

The qualitative X-ray diffraction (XRD) analysis was carried out with a Phillips X-ray X’Pert Pro diffractometer (Almelo, Holland) using an X-ray tube with copper anode (λ CuKα = 1.54178 Ǻ) with the following operation conditions: 30 mA and 40 kV. Registration was performed with the step-scanning method in steps of 0.04° in a range of 10–140° (2θ). The results were analysed using X’Pert High Score Plus software (v. 3.0d) and the ICDD database—PDF5+.

The microstructure of the samples was observed using an optical microscope (OM) Olympus GX-51 (Tokyo, Japan) under 100× and 500× magnification and a scanning electron microscope (SEM) JEOL JSM-6480 (Tokyo, Japan) under 300×, 1000×, and 3000× magnification. Additionally, an energy-dispersive X-ray spectroscopy detector (SEM-EDS) (IXRF, Austin, USA) was used to conduct a surface chemical composition analysis.

Micromechanical tests were performed using a Micro Combi Tester—MCT^3^ (Anton Paar, Corcelles-Cormondrèche, Switzerland). Measurements were conducted according to the recommendations of ISO 14577 [20]. A Vicker’s indenter (V-M 86) was used under a maximum load of 500 mN (0.5 N), acquisition rate of 10 Hz, loading and unloading rate of 1000 mN/min each, and endurance time under a maximum load of 10 s. H_IT_ hardness and E_IT_ elastic modulus were determined by the Oliver–Pharr method [21].

Tribological tests were performed using a TRN Tribometer (Anton Paar, Corcelles-Cormondrèche, Switzerland) in linear reciprocating motion with Ringer’s solution lubrication. The studies were carried out in accordance with the recommendations of the VAMAS technical note [22] as well as ASTM G99 and ASTM G133 standards [23,24]. The test parameters were selected based on preliminary studies and were as follows: the load of 10 N at a frequency of 2 Hz, on a friction path of 10 mm length, with a maximum linear speed of 6.28 cm/s and final friction distance of 100 m. This study was conducted at a temperature of 21 ± 1 °C and humidity of 40 ± 5%. Zirconium oxide (ZrO_2_) balls with a 6 mm diameter were used as counter-samples to simulate the endoprosthesis working pair of acetabular cup and femoral head for potential application. After the tribological tests, the average surface area of the wear tracks was evaluated using a Surftest SJ-500 contact profilometer (Mitutoyo, Tokyo, Japan).

## 3. Results

### 3.1. XRD Phase Composition

**Figure 1:**
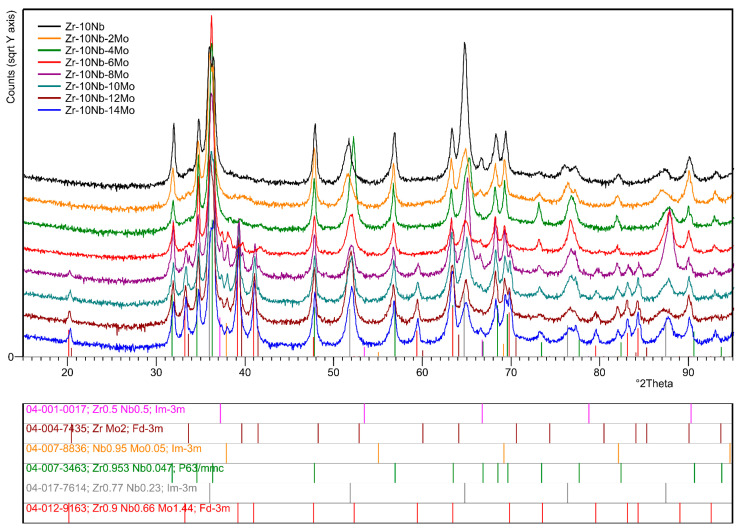
X-ray diffraction patterns of the studied samples.

The XRD method was used to analyse the phase composition of the studied materials and to identify their respective phases. Figure 1 presents the comparison of diffractograms obtained for the studied samples. Quantitative analysis was conducted using the Rietveld method. The results of the performed calculations are presented in Table 2 and Table 3.

For reference sample Zr-10Nb, consisting only of Zr and Nb, two phases were identified as follows: Zr_0.77_Nb_0.23_ (ICDD PDF 04-017-7614) Im$\bar{3}$m and Zr_0.953_Nb_0.047_ (ICDD PDF 04-007-3463) P6_3_/mmc. The presence of these phases was confirmed in each successive sample. Their proportion varied because of changes in the chemical composition of the alloys. The addition of molybdenum in sample Zr-10Nb-2Mo promoted the appearance of the intermetallic phase—ZrMo_2_ (ICDD PDF 04-004-7435) Fd$\bar{3}$m—as well as theNb_0.95_Mo_0.05_ (ICDD PDF 04-007-8836) Im$\bar{3}$m phase.

The X-ray pattern of sample Zr-10Nb-6Mo revealed the presence of a new phase—Zr_0.5_Nb_0.5_ (ICDD PDF 04-001-0017) Im$\bar{3}$m. For sample Zr-10Nb-8Mo with 8 wt.% of Mo, a ternary Zr_0.9_Nb_0.66_Mo_1.44_ (ICDD PDF 04-012-9163) Fd$\bar{3}$m phase was identified. The last analysed Zr-10Nb-14Mo sample (Mo = 14 wt.%) showed no other changes in the identified phase composition. It can be concluded that the Zr_0.953_Nb_0.047_ phase is the only one with a hexagonal crystal structure, as the remaining phases shown in the analysis crystallise in regular cells.

According to the detailed analysis, it can be noticed that both lattice parameters and the proportion of individual phases identified for the samples changed (Table 2 and Table 3). The phase with the least or non-present changes in the lattice parameters is ZrMo_2_, for which a_0_ = 0.7548 nm, according to the ICDD database. In contrast, the other identified phases revealed variations in these values because of differences in the atomic radii of the alloying elements (Zr = 0.206 nm, Nb = 0.198 nm, Mo = 0.190 nm). The distortions of the lattice parameters in the form of the displacement of X-ray patterns compared with the reference data can also be seen in Figure 1. This demonstrates the lattice expansion due to the formation of phases from differently sized atoms. For example, zirconium and niobium make up a substitional solid solution, in which the replacement of an element with a mismatching atomic radius results in a distortion of the lattice and a consequent change in the lattice parameter of the phase. Such a phenomenon can be well observed for the Zr_0.77_Nb_0.23_ and Zr_0.953_Nb_0.047_ phases. Additionally, the rising molybdenum content in the alloys contributes to these changes by further expanding the lattice, thus generating stress. As stated before, the Zr_0.953_Nb_0.047_ phase is the only one with a hexagonal crystal structure; thus, two parameters (a_0_ and c_0_) were calculated. Fluctuations in the values were observed for both of them, but for c_0_, they were greater. For example, for the sample with Mo = 6 wt.% the difference in c_0_ compared with the ICDD data reached 0.0050 nm, while for a_0_, it was 0.0036 nm.

The proportion of respective phases changed when the molybdenum content in the alloys was increased. This can be clearly seen for the Zr_0.77_Nb_0.23_ and Zr_0.953_Nb_0.047_ phases found in every sample. The increase in Mo promoted the formation of other phases, which caused a reduction in their contents with each successive sample. For sample Zr-10Nb-2Mo (Mo = 2 wt.%), an intermetallic phase—ZrMo_2_—formed, but increasing the Mo contents had a minor impact on its contribution up to the sample with Mo = 12 wt.%. For the last studied sample, the content of the ZrMo_2_ phase dropped by 50% in comparison with sample Zr-10Nb-2Mo. The amount of the Zr_0.9_Nb_0.66_Mo_1.44_ ternary phase exhibited an upward trend, reaching its high at 33.2(4) wt.% for sample Zr-10Nb-14Mo, with a successive and significant reduction in the phase content of the Zr_0.77_Nb_0.23_ and Zr_0.953_Nb_0.047_ phases.

### 3.2. Microstructure Analysis

**Figure 2:**
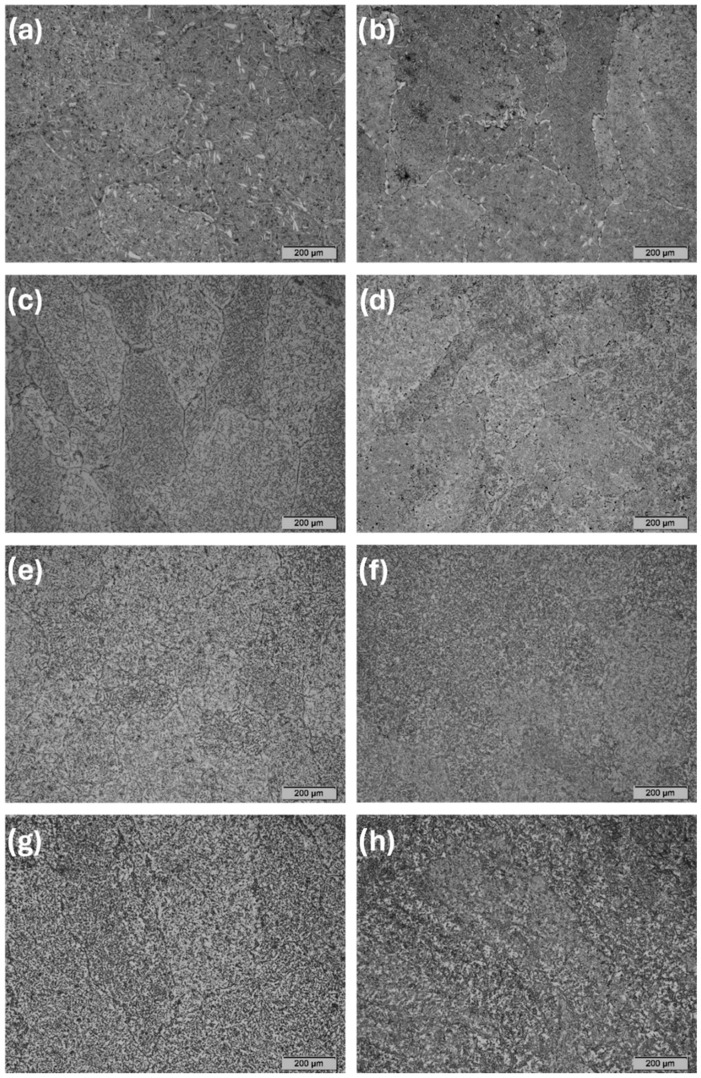
OM images (100×) of samples: (**a**) Zr-10Nb, (**b**) Zr-10Nb-2Mo, (**c**) Zr-10Nb-4Mo, (**d**) Zr-10Nb-6Mo, (**e**) Zr-10Nb-8Mo, (**f**) Zr-10Nb-10Mo, (**g**) Zr-10Nb-12Mo, and (**h**) Zr-10Nb-14Mo.

**Figure 3:**
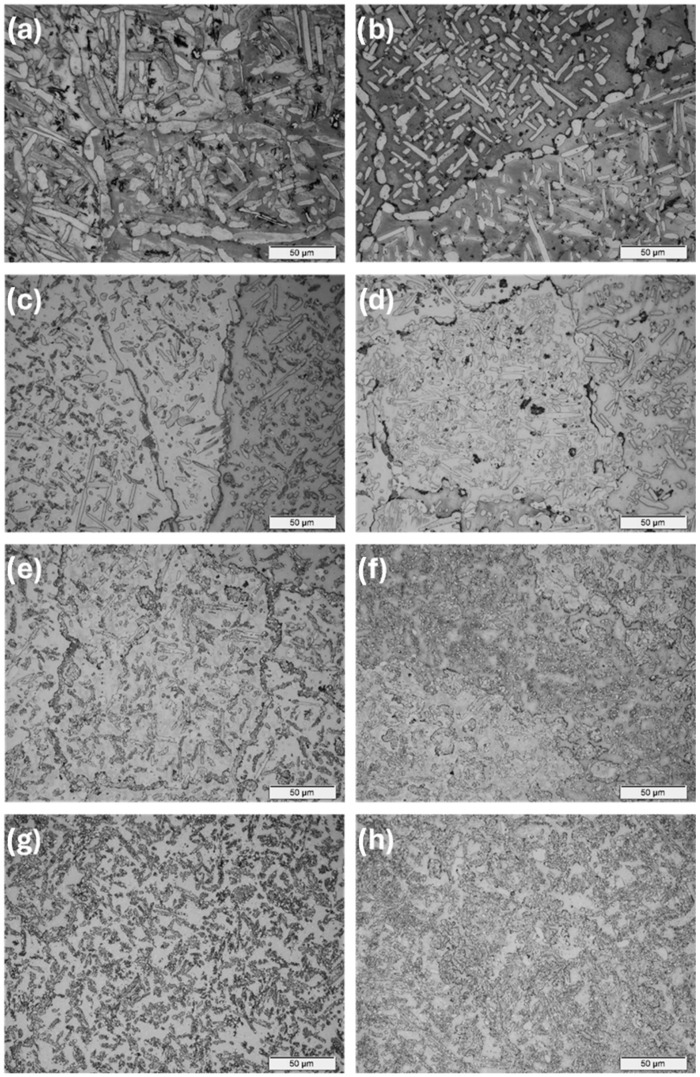
OM images (500×) of samples: (**a**) Zr-10Nb, (**b**) Zr-10Nb-2Mo, (**c**) Zr-10Nb-4Mo, (**d**) Zr-10Nb-6Mo, (**e**) Zr-10Nb-8Mo, (**f**) Zr-10Nb-10Mo, (**g**) Zr-10Nb-12Mo, and (**h**) Zr-10Nb-14Mo.

**Figure 4:**
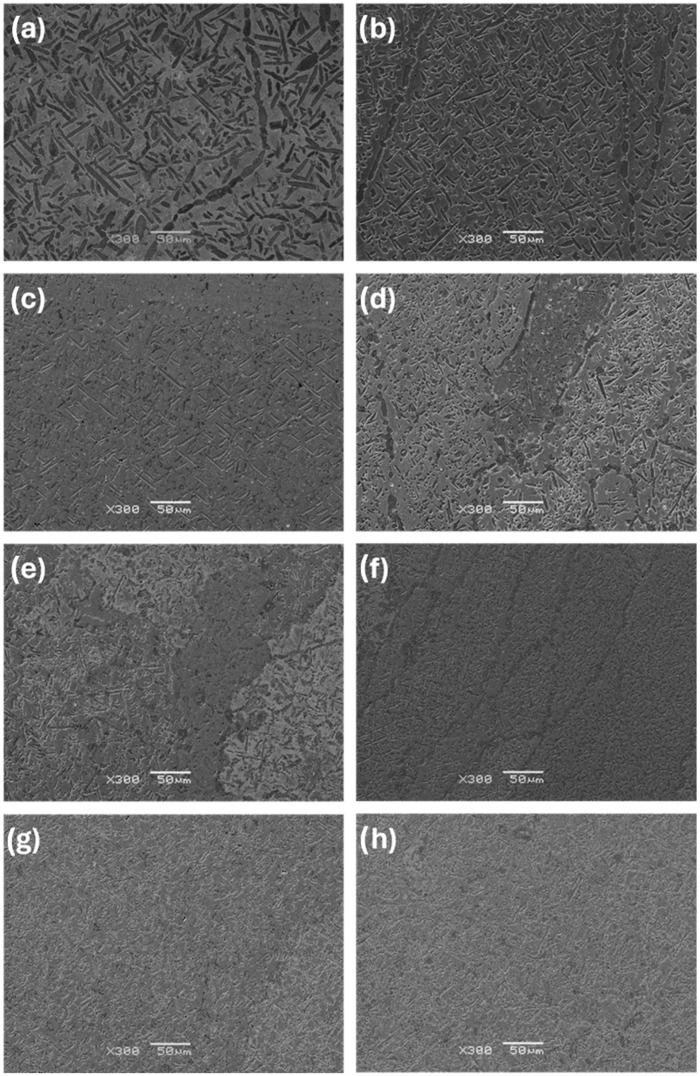
SEM images (300×) of samples: (**a**) Zr-10Nb, (**b**) Zr-10Nb-2Mo, (**c**) Zr-10Nb-4Mo, (**d**) Zr-10Nb-6Mo, (**e**) Zr-10Nb-8Mo, (**f**) Zr-10Nb-10Mo, (**g**) Zr-10Nb-12Mo, and (**h**) Zr-10Nb-14Mo.

**Figure 5:**
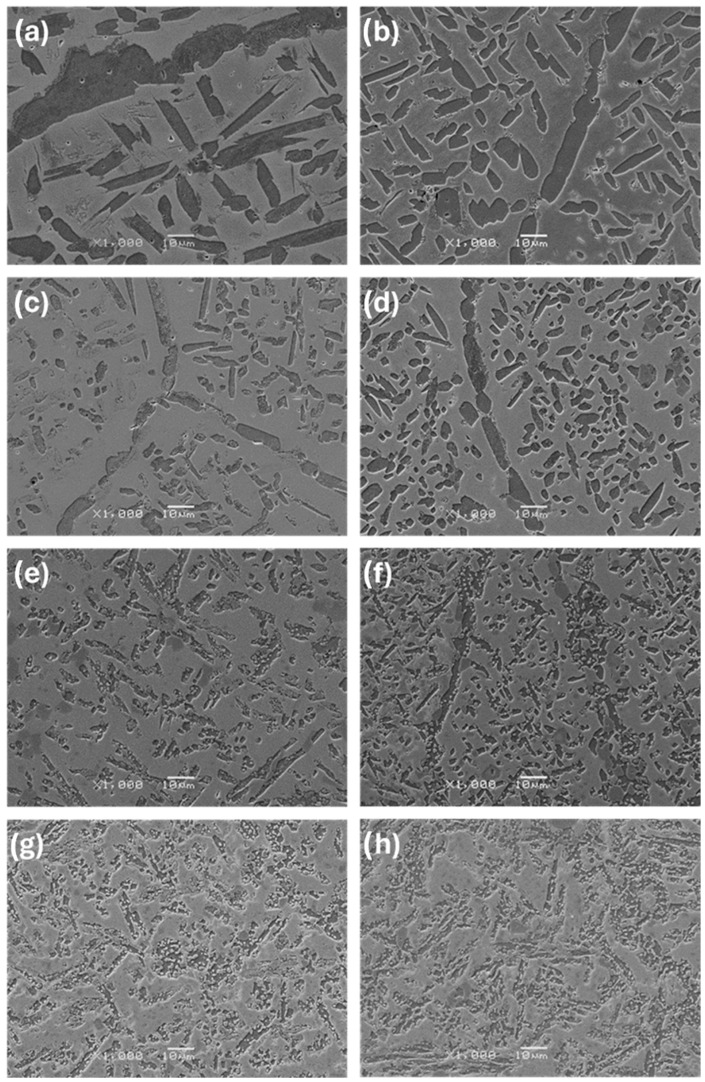
SEM images (1000×) of samples: (**a**) Zr-10Nb, (**b**) Zr-10Nb-2Mo, (**c**) Zr-10Nb-4Mo, (**d**) Zr-10Nb-6Mo, (**e**) Zr-10Nb-8Mo, (**f**) Zr-10Nb-10Mo, (**g**) Zr-10Nb-12Mo, and (**h**) Zr-10Nb-14Mo.

**Figure 6:**
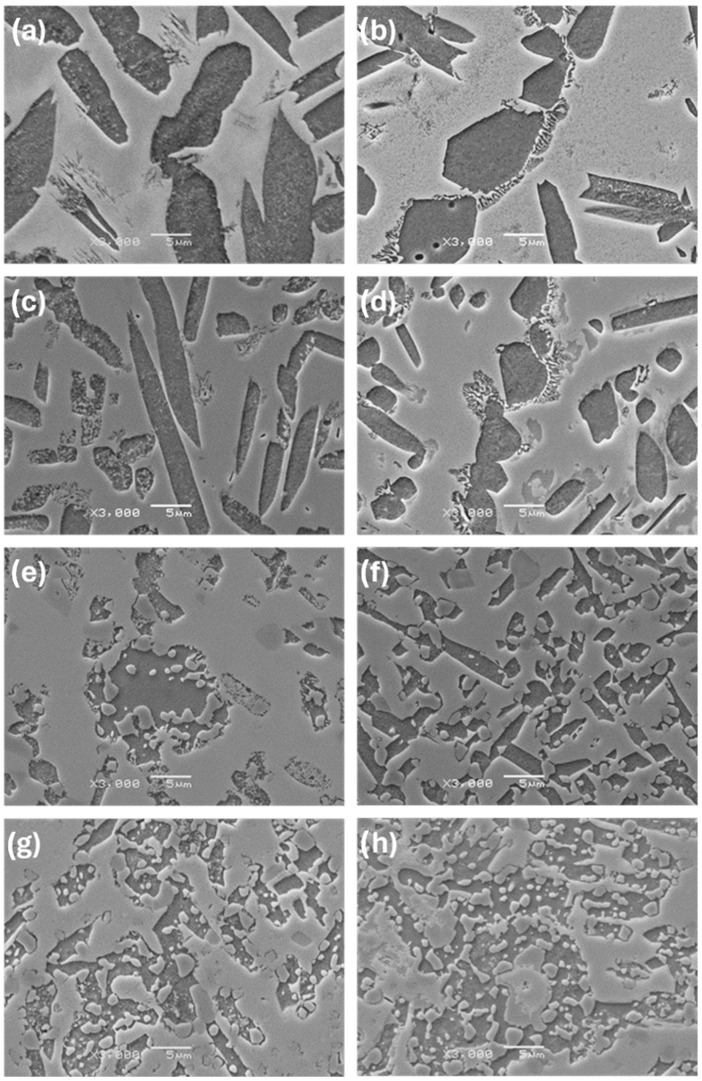
SEM images (3000×) of samples: (**a**) Zr-10Nb, (**b**) Zr-10Nb-2Mo, (**c**) Zr-10Nb-4Mo, (**d**) Zr-10Nb-6Mo, (**e**) Zr-10Nb-8Mo, (**f**) Zr-10Nb-10Mo, (**g**) Zr-10Nb-12Mo, and (**h**) Zr-10Nb-14Mo.

The observations carried out using the optical microscope OM (Figure 2 and Figure 3) and the scanning electron microscope SEM (Figure 4, Figure 5 and Figure 6) showed a varied microstructure of each tested sample. The presence of distinct elongated columnar and lamellar grains and near circular-shaped grains was determined. An arrangement of individual grains adopted different orientations with respect to each other. The shape of grains depended on the area of study caused by the solidification pattern, which is characteristic of the utilised production method (vacuum arc melting).

A grain decoration at the boundaries was observed in the samples containing from 0 to 6 Mo (wt.%), and for the subsequent alloys with increasing Mo content, it gradually became less evident. The shape of these grains changed through elongation because of the condensation of initially circular structures, which is particularly visible in the image of sample Zr-10Nb-2Mo (Figure 3). For samples visible in images (e) and (f) (Figure 6), the presence of big grains, consisting of smaller circular ones, was determined. The intensity of this phenomenon increased along with the molybdenum content in the samples.

Molybdenum inhibits grain growth, resulting in a progressive refinement and homogenisation of microstructure as its content increases [25]. As Chun et al. stated, molybdenum suppresses abnormal Zr-Nb grain growth by both stabilising the β-phase and causing a solute-dragging effect [26]. These changes were observed in consecutive images of the samples, with the greatest refinement and highest homogeneity in the microstructure found in sample Zr-10Nb-14Mo (Figure 3 and Figure 4).

### 3.3. EDS Elemental Distribution Analysis

**Figure 7:**
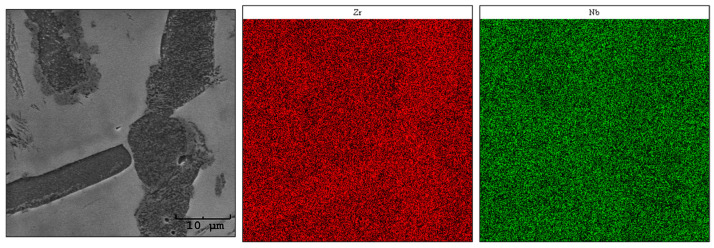
Maps of the chemical distribution of Zr and Nb (mass fraction)—sample Zr-10Nb.

**Figure 8:**
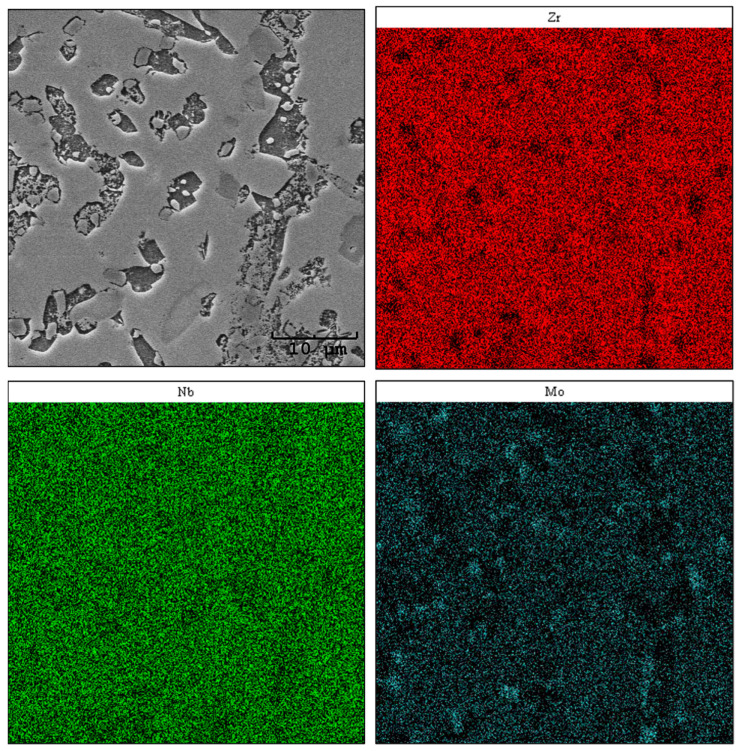
Maps of the chemical distribution of Zr, Nb, and Mo (mass fraction)—sample Zr-10Nb-8Mo.

**Figure 9:**
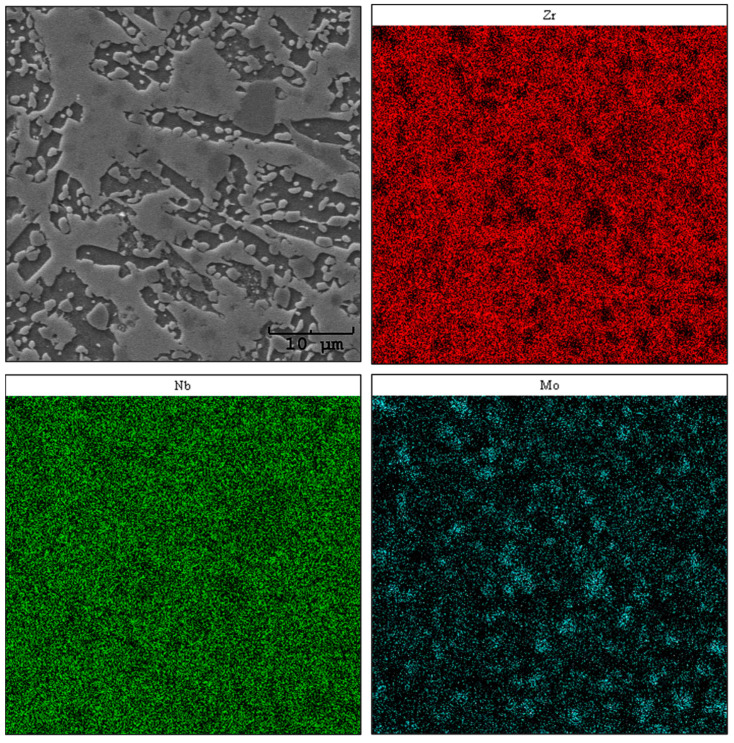
Maps of the chemical distribution of Zr, Nb, and Mo (mass fraction)—sample Zr-10Nb-14Mo.

Using the SEM-EDS method, an analysis of the elemental distribution in the areas of chosen samples was carried out. The obtained images are presented in Figure 7, Figure 8 and Figure 9. When interpreting the results of the chemical composition analysis by EDS, the micro-scale must be taken into consideration [27].

The analysis revealed an uneven distribution of zirconium and niobium over the investigated area of sample Zr-10Nb (Figure 7). The darker grains visible in the SEM image are slightly enriched in zirconium content, which may correspond to the Zr_0.953_Nb_0.047_ (04-007-3463) P6_3_/mmc phase, identified by X-ray diffraction. Regarding Roux and Vignes’s work [28], transition metals demonstrate the valence effect, thus increasing the diffusion rate along with an atomic number. The differences in concentration might also be an outcome of the differences in Zr (~1852 °C) and Nb (~2477 °C) melting points [29].

For sample Zr-10Nb-8Mo, the analysis revealed an uneven distribution of niobium and zirconium (Figure 8). In addition, minor aggregations of molybdenum were observed. Simultaneously, these areas are depleted in zirconium and overlap with the fine circular grains visible in the SEM microphotograph. The remaining regions show a uniform distribution, which, in terms of the performed phase composition study (XRD), may indicate the occurrence of a ternary phase [Zr_0.9_Nb_0.66_Mo_1.44_ (ICDD PDF 04-012-9163) Fd$\bar{3}$m] with mutual solubility of Zr, Nb, and Mo.

The analysis of the sample with 14% Mo (wt.%) showed an uneven distribution of Nb and areas with an uneven distribution of Zr and Mo (Figure 9). Similar to sample Zr-10Nb-8Mo, aggregations for the molybdenum map were observed, but their number increased. By looking at the microstructure shown in the SEM image, it may be concluded that a significant Mo content is located in small, circular grains, from which larger, elongated grains are comprised. As Prasad et al. stated [29], molybdenum requires a higher migration energy than the other researched elements in the presented study. The small circular aggregations might correspond to an intermetallic phase—ZrMo_2_ (ICDD PDF 04-004-7435) Fd$\bar{3}$m [30].

### 3.4. Micromechanical Properties

**Figure 10:**
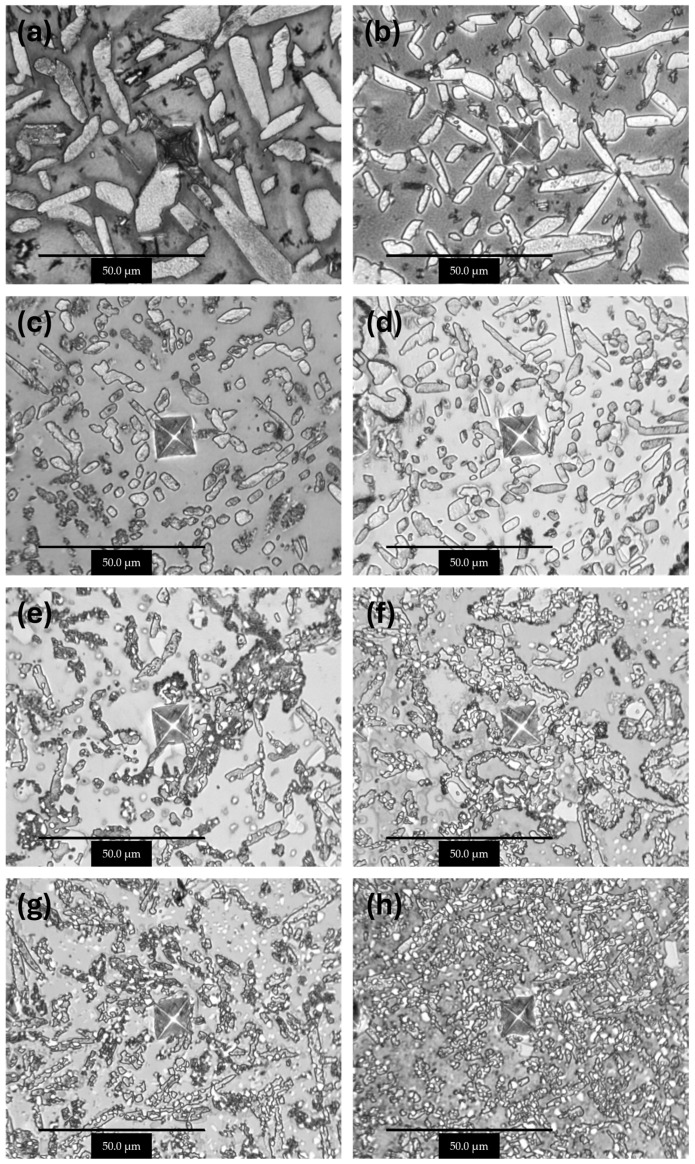
Microphotographs of indents in samples: (**a**) Zr-10Nb, (**b**) Zr-10Nb-2Mo, (**c**) Zr-10Nb-4Mo, (**d**) Zr-10Nb-6Mo, (**e**) Zr-10Nb-8Mo, (**f**) Zr-10Nb-10Mo, (**g**) Zr-10Nb-12Mo, and (**h**) Zr-10Nb-14Mo.

The micromechanical evaluation was conducted using a Micro Combi Tester, which was used to perform sets of indentations in the studied alloys. Images of the respective samples are shown in Figure 10. The values presented in Table 4 include the indentation modulus (E_IT_), indentation hardness (H_IT_), and Vickers indentation hardness (HV_IT_) calculated via the Oliver–Pharr method.

The performed measurements indicate that the value of the HV_IT_ parameter increased along with the molybdenum content in the samples, reaching its highest value for the last sample (Mo = 14 wt.%). Microhardness increased by more than 60%—from 2.8 GPa to 4.5 GPa. 

The results are subject to certain deviations from the average values. The microscale of the conducted studies and surface roughness can be considered one of the reasons [31]. The lowest indentation modulus (E_IT_), indentation hardness (H_IT_), and Vickers indentation hardness (HV_IT_) were measured for sample Zr-10Nb, consisting of only Zr and Nb. An increase of almost 30% was recorded for the elastic modulus, starting from 78.8 GPa and reaching 102.2 GPa ultimately. The acquired results may lead to the conclusion that the molybdenum content greatly affects the mechanical properties of studied alloys [32,33].

Images of indentations shown in Figure 10 present areas of the conducted tests. The indents made in all samples, excluding sample Zr-10Nb, resemble each other in terms of shape, having uneven, irregular edges. The morphology of the microindentation marks also confirms that the alloy’s resistance to permanent deformation increases along with molybdenum content. A reduction in the surface area and depth of the indentations can be observed, which is directly related to the improvement in the micromechanical properties.

### 3.5. Tribological Behaviour

**Figure 11:**
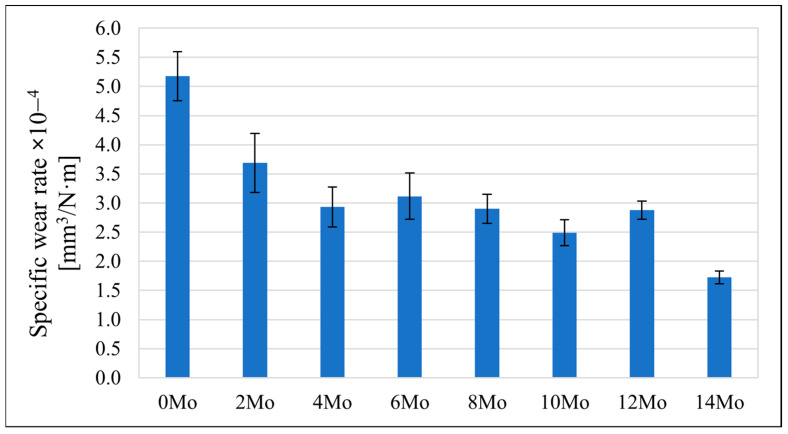
The specific wear rate (w_s_) of the tested samples.

**Figure 12:**
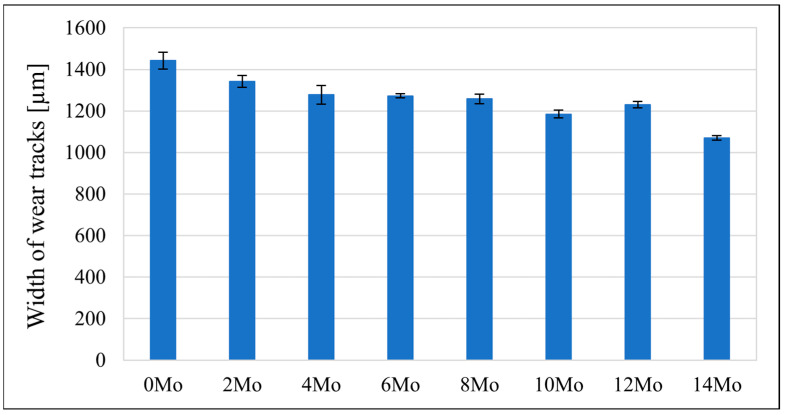
The width of wear tracks (W) measured for the samples.

**Figure 13:**
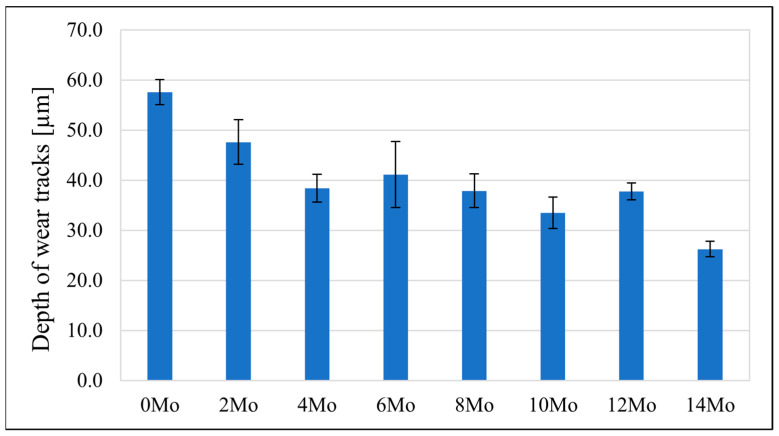
The depth of wear tracks (d) measured for the samples.

**Figure 14:**
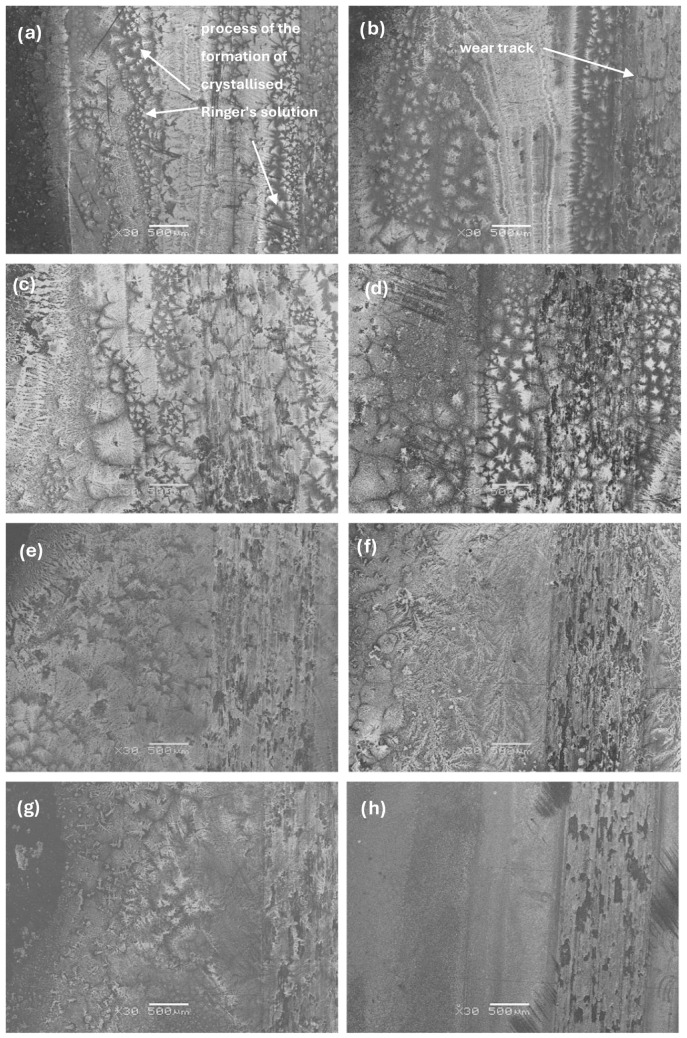
SEM images of wear tracks before ultrasound cleaning for samples: (**a**) Zr-10Nb, (**b**) Zr-10Nb-2Mo, (**c**) Zr-10Nb-4Mo, (**d**) Zr-10Nb-6Mo, (**e**) Zr-10Nb-8Mo, (**f**) Zr-10Nb-10Mo, (**g**) Zr-10Nb-12Mo, and (**h**) Zr-10Nb-14Mo.

**Figure 15:**
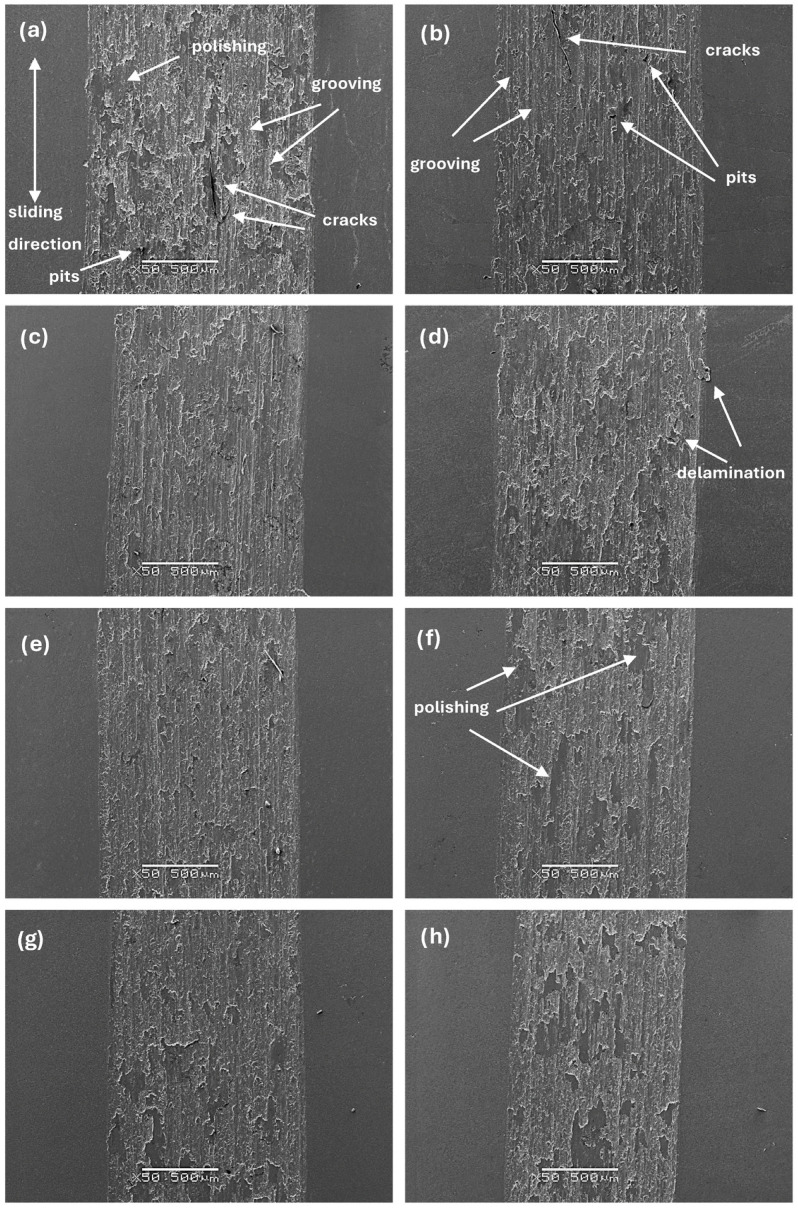
SEM images of wear tracks after ultrasound cleaning for samples: (**a**) Zr-10Nb, (**b**) Zr-10Nb-2Mo, (**c**) Zr-10Nb-4Mo, (**d**) Zr-10Nb-6Mo, (**e**) Zr-10Nb-8Mo, (**f**) Zr-10Nb-10Mo, (**g**) Zr-10Nb-12Mo, and (**h**) Zr-10Nb-14Mo.

**Figure 16:**
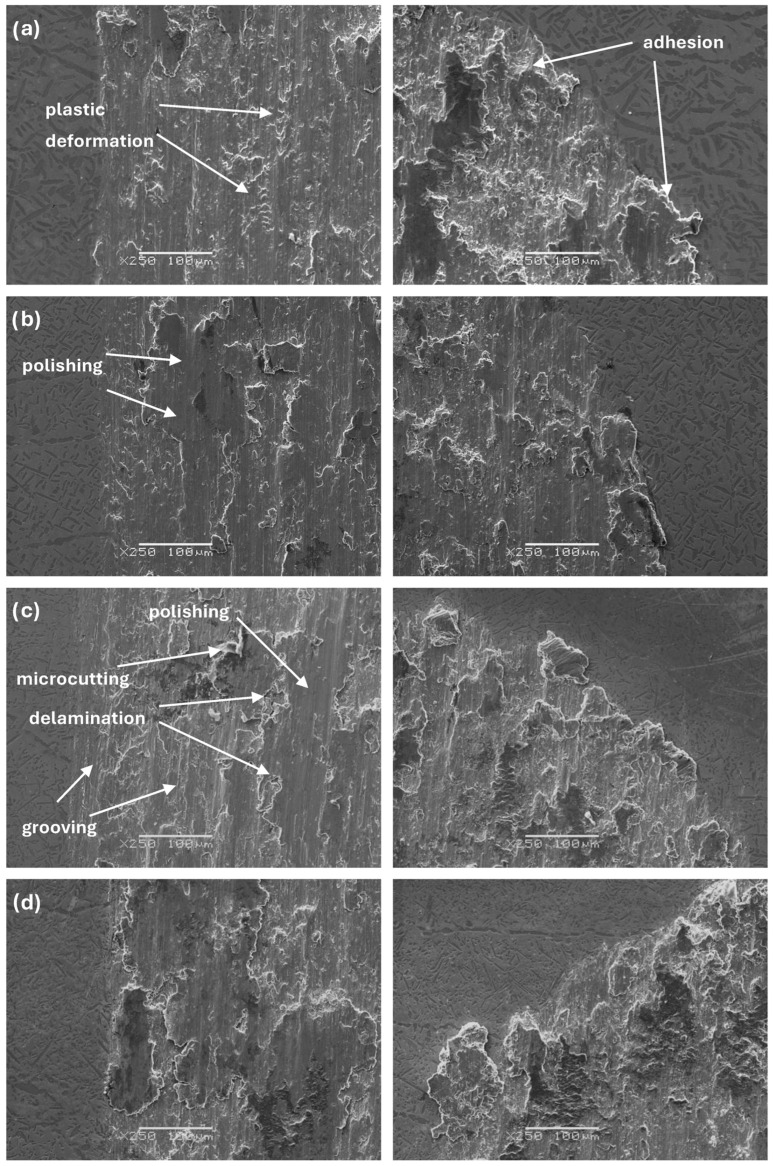

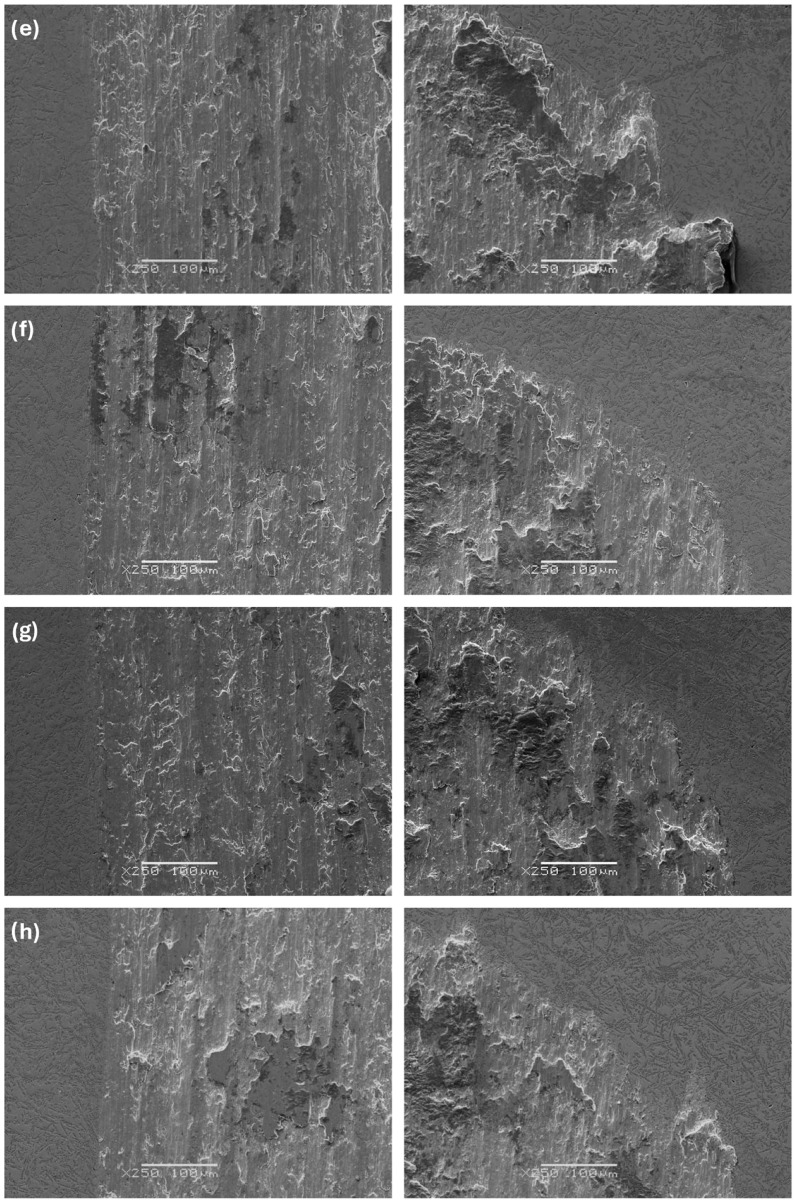
SEM images of the beginning and edge of the wear track of samples: (**a**) Zr-10Nb, (**b**) Zr-10Nb-2Mo, (**c**) Zr-10Nb-4Mo, (**d**) Zr-10Nb-6Mo, (**e**) Zr-10Nb-8Mo, (**f**) Zr-10Nb-10Mo, (**g**) Zr-10Nb-12Mo, and (**h**) Zr-10Nb-14Mo.

**Figure 17:**
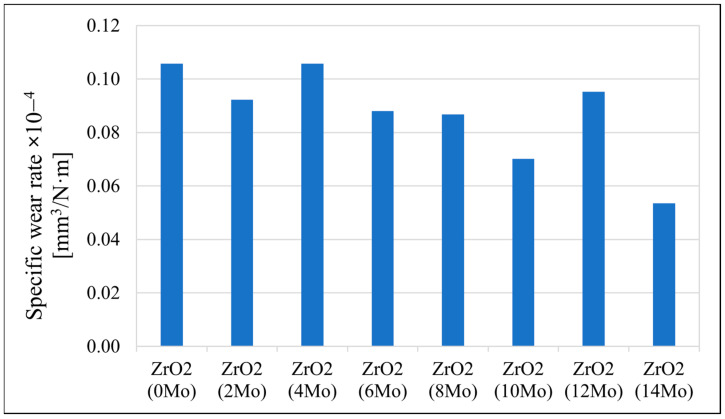
Specific wear rates (w_s_) of the ZrO_2_ counter-samples.

The sample’s tribological behaviour was assessed using a TRN Tribometer in a linear reciprocating motion. Ringer’s solution was used as a lubricant, simulating the body’s extracellular fluid environment. In addition, counter-samples were made of ZrO_2_, a well-known medical ceramic used in hard tissue surgery. The tribological parameters measured for the alloys, including specific wear rate—w_s_, wear track width—W, and depth—d, are presented in Figure 11, Figure 12 and Figure 13. The SEM microphotographs of wear tracks before and after ultrasound cleaning are shown in Figure 14, Figure 15 and Figure 16. As for the counter-samples, the measured wear rate is shown in Figure 17.

Tribological tests in linear reciprocating motion showed that the initial alloy Zr-10Nb was characterised by significant wear (specific wear rate w_s_—5.2∙10^−4^ mm^3^/Nm; track width W—1440 μm; track depth d—58 μm). With increasing molybdenum content (from 2–14 wt.%), a reduction of approximately 30 to 70% in wear, a 30% reduction in the width of the abrasion track, and a more than 40% reduction in the depth of the track could be observed. The most favourable results were obtained for sample Zr-10Nb-14Mo (specific wear rate w_s_—1.7∙10^−4^ mm^3^/Nm; trace width W—1070 μm, depth d—about 26 μm). The improvement in the tribological properties of the alloy is directly related to the modification of the structure induced by molybdenum doping. The progressive grain refinement and homogenisation of the microstructure directly affect the increase in mechanical properties such as H_IT_ hardness and Young’s modulus E_IT_ observed during instrumental microindentation tests. As a result of the increase in these properties, there is a simultaneous significant reduction in wear. This has a major impact on the extension of the service life of the alloys meant for endoprosthetic reconstruction.

Analysis of the morphology of wear tracks before ultrasound cleaning (Figure 14) shows the process of the formation of crystallised Ringer’s solution at the wear track boundaries. For samples Zr-10Nb and Zr-10Nb-4Mo, cracks propagating through the salt crystals are visible. The intensity of the salt crystallisation at the boundary of the ball friction path decreased with increasing molybdenum content in the samples, which is also associated with a significant smoothing of the friction surface, characterised by a reduction in the depth and width of the observed wear tracks.

The microphotographs of the wear tracks after the cleaning process (Figure 15 and Figure 16), in the middle and at the end of the wear track specifically, allow us to identify the main wear mechanisms occurring during tribological cooperation of the alloys and zirconium oxide (ZrO_2_). As previously discussed, the specific wear rate largely depends on the doping of molybdenum, as well as the morphology and properties of the alloy that change as a result. It can be concluded that the tribological wear of the studied materials occurs mainly according to adhesive and abrasive wear mechanisms [34].

The reduction in wear of the tribological counterpart in the form of ZrO_2_ balls is associated with the improvement in mechanical properties and the reduction in wear of the alloys, which, when worn to a lesser extent, generate fewer wear particles acting as an additional abrasive (third body), causing additional wear on the balls and the alloys.

**Figure 18:**
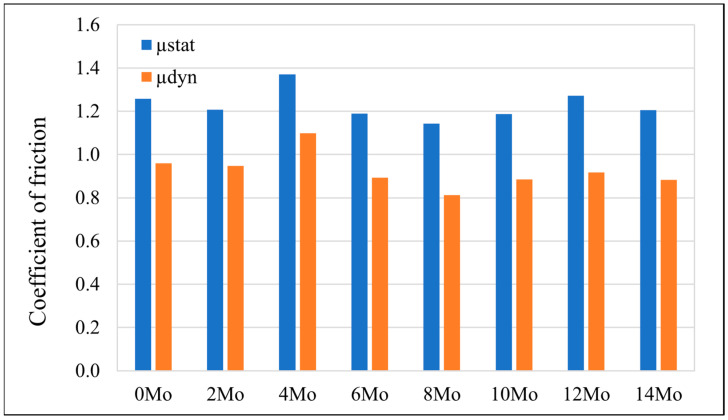
Static and dynamic friction ratio of the tested samples.

As a result of the performed measurements, the static and dynamic friction ratio was determined for each sample. The findings are presented in Figure 18.

Sample Zr-10Nb-8Mo presented the lowest friction ratio of µ_stat_ = 1.143; µ_dyn_ = 0.812, whereas sample Zr-10Nb-4Mo reached the highest value of µ_stat_ = 1.370; µ_dyn_ = 1.098. During reciprocating motion, mixed friction occurs, in this case, with a predominance of the less favourable boundary friction over the fluid friction, hence, the high values of the COFs measured for samples. Under dry friction, friction heat might cause an oxidation of certain alloying elements, resulting in an increase in surface roughness [35].

## 4. Discussion

According to the results of the conducted research, the obtained Zr-Nb-Mo alloys exhibit particularly interesting properties, especially regarding their mechanical and tribological characteristics, paving the way for a better understanding and further studies of these novel materials.

The molybdenum content in the alloy significantly affects its properties, contributing to changes such as grain refinement or an increase in microhardness HV_IT_, which agrees with the published literature data [25]. Both of these features show a significant influence on the tribological properties of the alloys, contributing to their improvement [34,35]. In comparison with well-known biomaterials, such as AISI 316L SS or Ti-6Al-4V, the proposed alloys perform well, indicating their exceptional application potential [36,37]. The E_IT_ parameter measured for the alloys presented in this paper increased along the Mo content, reaching its maximum value around 102 GPa, which is higher than human bone, yet still lower than the values measured for commonly recognised CoCr (~200 GPa) or Ti-6Al-4V (~110 GPa) [38,39]. The 8 wt.% content of Mo promotes the formation of a ternary Zr_0.9_Nb_0.66_Mo_1.44_ (ICDD PDF 04-012-9163) Fd$\bar{3}$m phase, which might correspond with the areas rich in Mo, as can be seen in EDS mapping results (Figure 8). In addition, Zr, Nb, and Mo belong to the transition metals group, so the diffusion rate increases with their atomic numbers [28], which can also be considered one of the reasons for such uneven distribution. The content of this ternary phase increased along with molybdenum addition, reaching 33.2 wt.% for sample Zr-10Nb-14Mo.

The requirements for biomaterials are broad, so we suggest extending the characterisation of the Zr-Nb-Mo alloys, with a focus on biological tests and possible further processing of the mechanical parameters.

## 5. Conclusions

Combining powder metallurgy (PM) and vacuum arc melting (VAM) methods made it possible to obtain ternary alloys of the Zr-Nb-Mo system. The presented results allow the following conclusions to be drawn:

- The produced melts are multiphase with mutual solubility of the elements comprising the material.
- Microscopic analysis showed that the higher molybdenum content in the alloys favoured the formation of a more refined and homogeneous microstructure.
- EDS analysis showed a slight concentration of elements in the form of areas enriched in individual elements. In particular, areas of uneven mutual distribution for zirconium and molybdenum were present.
- Increasing the molybdenum content in the samples contributed to an enhancement in the micromechanical and tribological properties.
- The wear of the studied alloys occurs according to abrasive and adhesive mechanisms, with an increasing amount of Mo improving the tribological performance.

## Figures and Tables

**Table 1:** Compositions of the studied samples.

Sample Identification	Element Content [wt.%]		
	Zr	Nb	Mo
Zr-10Nb	90	10	0
Zr-10Nb-2Mo	88	10	2
Zr-10Nb-4Mo	86	10	4
Zr-10Nb-6Mo	84	10	6
Zr-10Nb-8Mo	82	10	8
Zr-10Nb-10Mo	80	10	10
Zr-10Nb-12Mo	78	10	12
Zr-10Nb-14Mo	76	10	14

**Table 2:** Quantitative XRD results for studied alloys using the Rietveld method (lattice parameters).

Phase		ICDD	Sample (Mo wt.%)							
			0%	2%	4%	6%	8%	10%	12%	14%
		Lattice Parameters [nm]								
Zr_0.77_Nb_0.23_	a_0_	0.3524	0.3536(1)	0.3530(1)	0.3511(1)	0.3522(1)	0.3515(1)	0.3520(1)	0.3525(1)	0.3525(1)
Zr_0.953_Nb_0.047_	a_0_	0.3216	0.3246(1)	0.3247(1)	0.3248(1)	0.3252(1)	0.3246(1)	0.3248(1)	0.3246(1)	0.3247(1)
	c_0_	0.5134	0.5175(1)	0.5177(1)	0.5178(1)	0.5184(1)	0.5176(1)	0.5177(1)	0.5177(1)	0.5176(1)
ZrMo_2_	a_0_	0.7548	-	0.7548(1)	0.7548(1)	0.7547(1)	0.7548(1)	0.7547(1)	0.7548(1)	0.7548(1)
Nb_0.95_Mo_0.05_	a_0_	0.3294	-	0.3294(1)	0.3295(1)	0.3334(1)	0.3346(1)	0.3350(1)	0.3351(1)	0.3350(1)
Zr_0.5_Nb_0.5_	a_0_	0.3447	-	-	-	0.3447(1)	0.3407(1)	0.3407(1)	0.3407(1)	0.3407(1)
Zr_0.9_Nb_0.66_Mo_1.44_	a_0_	0.7610	-	-	-	-	0.7612(2)	0.7626(2)	0.7628(2)	0.7629(2)

**Table 4:** Measured micromechanical parameters (E_IT_, H_IT_, HV_IT_).

Tested Samples	E_IT_ [GPa]	H_IT_ [GPa]	HV_IT_
Zr-10Nb	78.8 ± 4.6	2.8 ± 0.4	261.1 ± 35.3
Zr-10Nb-2Mo	83.4 ± 2.1	3.0 ± 0.1	287.3 ± 9.8
Zr-10Nb-4Mo	89.7 ± 3.2	3.5 ± 0.3	329.6 ± 24.7
Zr-10Nb-6Mo	88.0 ± 1.7	3.4 ± 0.1	324.2 ± 10.7
Zr-10Nb-8Mo	98.7 ± 3.5	3.9 ± 0.2	364.6 ± 15.7
Zr-10Nb-10Mo	97.6 ± 5.0	3.9 ± 0.2	370.9 ± 21.0
Zr-10Nb-12Mo	100.1 ± 3.2	4.1 ± 0.2	391.6 ± 20.4
Zr-10Nb-14Mo	102.2 ± 4.6	4.5 ± 0.4	429.2 ± 41.4

**Table 3:** Quantitative XRD results for studied alloys using the Rietveld method (contents).

Phase	Sample (Mo wt.%)							
	0%	2%	4%	6%	8%	10%	12%	14%
	Content [wt.%]							
Zr_0.77_Nb_0.23_	49.5(2)	49.5(2)	49.7(2)	49.2(2)	45.5(2)	39.4(2)	36.4(2)	32.1(5)
Zr_0.953_Nb_0.047_	50.5(2)	49.5(2)	49.3(2)	47.1(2)	41.1(2)	36.3(2)	34.2(2)	31.2(1)
ZrMo_2_	-	0.6(1)	0.5(1)	0.6(1)	0.5(1)	0.5(1)	0.4(1)	0.3(1)
Nb_0.95_Mo_0.05_	-	0.4(1)	0.5(1)	2.6(1)	2.6(1)	2.8(1)	3.0(1)	2.7(1)
Zr_0.5_Nb_0.5_	-	-	-	0.5(1)	1.4(1)	1.3(1)	0.9(1)	0.5(1)
Zr_0.9_Nb_0.66_Mo_1.44_	-	-	-	-	12.9(1)	19.7(1)	21.1(1)	33.2(4)

## Data Availability

The original contributions presented in the study are included in the article, further inquiries can be directed to the corresponding authors.
